# Response Surface Methodology to Optimize Enzymatic Preparation of Deapio-Platycodin D and Platycodin D from Radix Platycodi

**DOI:** 10.3390/ijms13044089

**Published:** 2012-03-28

**Authors:** Wei Li, Li-Chun Zhao, Zi Wang, Yi-Nan Zheng, Jian Liang, Hui Wang

**Affiliations:** 1 College of Chinese Medicinal Materials, Jilin Agricultural University, Changchun 130118, China; E-Mails: liwei7727@126.com (W.L.); wangzi8020@126.com (Z.W.); zhenyinan@tom.com (Y.-N.Z.); 2 The Affiliated Ruikang Hospital of Guangxi Traditional Chinese Medical College, Nanning 530011, China; E-Mail: hyzlc@126.com; 3 China-Japan Union Hospital, Jilin University, Changchun 130033, China

**Keywords:** snailase, deapio-platycodin D, platycodin D, radix platycodi, response surface methodology

## Abstract

In the present work, we reported the enzymatic preparation of deapio-platycodin D (dPD) and platycodin D (PD) optimized by response surface methodology (RSM) from Radix Platycodi. During investigation of the hydrolysis of crude platycosides by various glycoside hydrolases, snailase showed a strong ability to transform deapio-platycoside E (dPE) and platycoside E (PE) into dPD and PD with 100% conversion. RSM was used to optimize the effects of the reaction temperature (35–45 °C), enzyme load (5–20%), and reaction time (4–24 h) on the conversion process. Validation of the RSM model was verified by the good agreement between the experimental and the predicted values of dPD and PD conversion yield. The optimum preparation conditions were as follows: temperature, 43 °C; enzyme load, 15%; reaction time, 22 h. The biotransformation pathways were dPE→dPD3→dPD and PE→PD3→PD, respectively. The determined method may be highly applicable for the enzymatic preparation of dPD and PD for medicinal purposes and also for commercial use.

## 1. Introduction

**Figure 1:**
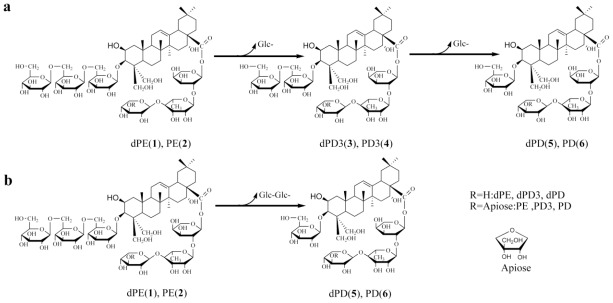
Biotransformation pathway for production of deapio-platycodin D (**5**) and platycodin D (**6**): (**a**), indirectly, in this case dPE (**1**) and PE (**2**) converted to dPD (**5**) and PD (**6**) with an intermediate dPD3 (**3**) and PD3 (**4**); (**b**), directly, in this case dPE (**1**) and PE (**2**) converted to dPD (**5**) and PD (**6**) with any intermediates.

Radix Platycodi (Jiegeng in Chinese), the root of *Platycodon grandiflorum* A. DC (Campanulaceae), is frequently used in Asian countries as a traditional medicine. The major pharmacologically active components in Jiegeng are a class of oleanane-type triterpenoid saponins, more commonly known as platycosides. Until now, more than 30 were discovered and identified in *P. grandiflorum*, mainly including deapio-platycodin D (dPD) and platycodin D (PD) [1,2]. Figure 1 shows the chemical structures of platycosides studied in this paper. Among these platycosides, PD as a major platycoside and marker reference substance, exhibited strong cytotoxicity against tumor cell lines and can be implemented as a therapeutic agent for the treatment of cancer [3–6]. Therefore, there is great interest in the preparation of platycosides (mainly including dPD and PD) in terms of their bioactivities and commercial use[7,8].

Recently, a variety of methods, including mild acid hydrolysis, alkali treatment and microbial conversion have been applied to the preparation of platycosides [9]. However, these chemical methods inevitably produced side-reactions and environmental pollution. Prior to this study, *Aspergillus niger* (*A. niger*) was used to convert platycosides, but these preparation processes were time-consuming, with low selectivity and low conversion rate [10]. As an alternative to the above preparation methods, enzymatic preparation (EP) has been implicated as the most promising for the preparation of active constituents via the selective hydrolysis of the sugar moieties, owing to its high specificity, yield and productivity [11–13]. Recently, snailase (a complex of cellulase, hemicellulase, pectinase and β-glucuronidase), extracted from the digestive tract of snails [14], have received increasing attention due to strong hydrolysis ability [15,16]. We have previously reported snailase effectively transformed 20(*S*)-protopanaxadiol-type ginsenosides to ginsenosides Rh1 via the cleavage of sugar moieties at the C-20 position [17]. During our investigations aiming to prepare large quantities of dPD and PD, snailase was found to be powerful for transforming dPE and PE to dPD and PD. Response surface methodology (RSM), an effective statistical technique, can use quantitative data to evaluate multiple parameters and their interactions by establishing a mathematical model [18,19]. Box-Behnken design (BBD), as one of the RSM designs, is more efficient and easier to arrange and interpret experiments in comparison with others, and widely applied in many studies [20,21]. Our research team has employed this technology for the extraction of chromones in Radix Saposhnikoviae [22].

The main objective of the present work was to investigate the feasibility of using enzymes in preparing dPD and PD via biotransformation of dPE and PE. To the best of our knowledge, snailase preparation of dPD and PD has not previously been published. Furthermore, the effects of reaction temperature, enzyme load, and reaction time on the enzymatic preparation efficiency and their interactions were also systemically analyzed for the first time using RSM and BBD methods.

## 2. Results and Discussion

### 2.1. Selection of Glycolytic Enzymes

To select enzymes for the concurrent bioconversion of platycosides in crude saponins to dPD and PD, the hydrolyzing ability of several glycolytic enzymes based on the glycosidic moiety at C-3 position, snailase, β-glucanase, cellulase and amylase, was evaluated. Individual kinetics of hydrolysis of 50 mg/mL crude platycosides were investigated incubating each glycolytic enzyme at 37 °C for 24 h. The enzyme amount was adjusted to 50 U/g of crude platycosides (0.1 U/mL). Although the above enzyme gave the same hydrolysis pattern and ability, complete hydrolysis to dPD and PD was only achieved in 24 h by snailase (data not shown). Therefore, snailase was selected for future investigations.

### 2.2. Model Fitting

After the preliminary ranges of the preparation variables were determined by one-factor-at-a-time experiments, the three independent variables, the reaction temperature (*X*_1_, 35–45 °C), enzyme load (*X*_2_, 5–20%) and reaction time (*X*_3_, 4–24 h), were fixed to optimize the yields of dPD and PD. The whole design consisted of 17 experimental points as listed in Table 1, and five replicates (run 13–17) at the center of the design were used to estimate an experimental error sum of squares. The triplicates were performed at all design points in randomized order.

As Table 2 shows, the analysis of variance (ANOVA) of conversion yield of dPD and PD indicated that experimental data had a determination coefficient (*R*^2^) of 0.977 with the calculated model with no significant lack of fit at *P* > 0.05. That means that the calculated model was able to explain 97.7% of the results. The results indicated that the model used to fit response variables was significant (*P* < 0.0001) and adequate to represent the relationship between the response and the independent variables. *F*-test suggested that model had a very high model *F*-value (*F* = 33.43), indicating this model was highly significant. *R*^2^_adj_ value (adjusted determination coefficient) is the correlation measure for testing the goodness-of-fit of the regression equation [23]. The *R*^2^_adj_ value of this model is 0.9880, which indicated only 1.2% of the total variations were not explained by the model. Meanwhile, a relatively lower value of coefficient of variation (*CV* = 4.35) showed a better precision and reliability of the experiments carried out [24].

It can be seen in Table 3 that conversion yield of dPD and PD was affected most significantly by reaction temperature (*X*_1_) (*P* < 0.0001), followed by reaction time (*X*_3_) (*P* < 0.0002) and enzyme load (*X*_2_) (*P* = 0.0016). It was evident that all the quadratic parameters (*X*_1_^2^, *X*_2_^2^, *X*_3_^2^) were significant at the level of *P* < 0.05 or *P* < 0.0001, whereas all the interaction quadratic parameters were insignificant (*P* > 0.1). Predicted response *Y* for the yield of dPD and PD could be expressed by the following second-order polynomial equation in terms of coded values.

$$Y=-106.38+5.19X_{1}+0.978X_{2}+0.18X_{3}-2.70X_{1}X_{2}+5.24X_{2}X_{3}-0.06\times 10^{-3}{X_{1}}^{2}-0.03{X_{2}}^{2}-5.92{X_{3}}^{2}$$

Where *Y* is the yield of dPD and PD (mg/mL), and *X*_1_, *X*_2_ and *X*_3_ are the coded variables for reaction temperature, enzyme load and reaction time, respectively.

### 2.3. Analysis of Response Surface

**Figure 2:**
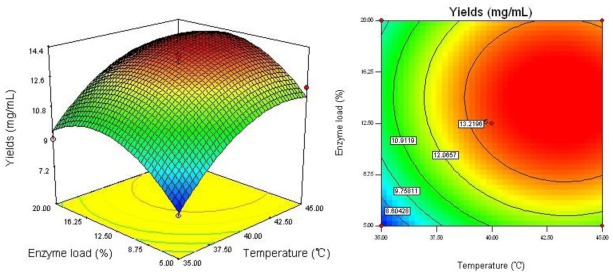
Response surface plot and contour plot of reaction temperature and enzyme load.

**Figure 3:**
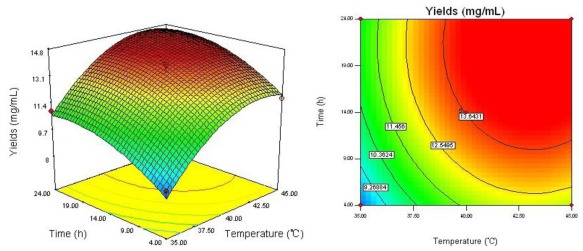
Response surface plot and contour plot of reaction temperature and reaction time.

**Figure 4:**
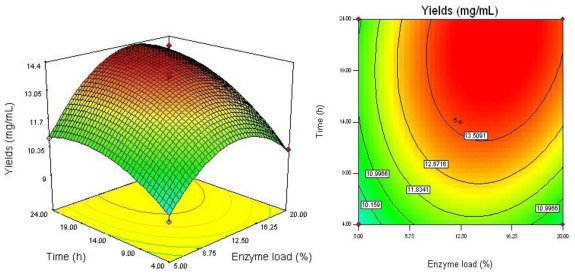
Response surface plot and contour plot of enzyme load and reaction time.

The regression equation was graphically represented by 3D response surface and 2D contour plots. From three dimensional response surface curves and contour plots shown in Figures 2–4, the effect of the independent variables and their mutual interaction on the yield of dPD and PD can be seen.

Figure 2 shows the interaction between reaction temperature (*X*_1_) and enzyme load (*X*_2_) on the yield of dPD and PD. Increase in reaction temperature from 35 to 45 °C with enzyme load from 5 to 20%, enhanced the conversion yield of dPD and PD. While with increase of reaction temperature over 45 °C there was a gradual decline in the response and enzyme load over 20% did not show any obvious effect on the yield of dPD and PD. It could be explained that, increasing reaction temperature may enhance snailase activity in preparation process.

The effect of combination of reaction temperature (*X*_1_) and reaction time (*X*_3_) on the yield of dPD and PD is shown in Figure 3. It may be also observed that increase of reaction temperature from 35 to 45 °C and reaction time from 4 to 22 h, the yield of dPD and PD was increasing gradually. However, this interactive effect of reaction time with reaction temperature on the yield of dPD and PD was not very significant (*P* = 1.00).

As shown in Figure 4 and Table 3, the interaction of enzyme load (*X*_2_) and reaction time (*X*_3_) had a much weaker effect on the yield of dPD and PD (*P* = 0.1064). It depicted that the highest conversion yield could be achieved when using about 15% of enzyme load and 22 h of reaction time. However, the conversion yield did not increase with the enzyme load over 15%. Moreover, 22 h of reaction time is enough for enzymatic preparation to convert all dPE and PE to dPD and PD.

### 2.4. Optimal Conditions and Model Verification

In this study, the aim of optimization was to find the conditions which gave the maximum conversion yield of dPD and PD. The software predicted the optimum reaction temperature, enzyme load and reaction time was 43.13 °C, 15% and 22.23 h, respectively. The software predicted the yield of dPD and PD was 14.93 mg/mL.

As shown in Table 4, three parallel experiments were carried out under the optimal conditions, and the average yield of dPD and PD was 14.81 mg/mL. Compared with the value predicted by Design Expert 7.1.6, the results showed that the predicted value was very close to the actual results. This indicated that the optimization is reliable in the present study.

### 2.5. LC/ESI-MS Analysis and Structures Elucidation

Mass spectrometry, especially MS with electrospray ionization, is a valuable analytical tool in term of providing information on the molecular weights of polar and thermally labile compounds as saponins [25,26].

**Figure 5:**
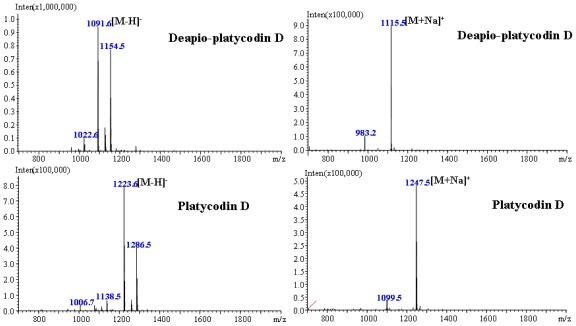
ESI–MS spectra of deapio-platycodin D and platycodin D with [M−H]^−^ and [M+Na]^+^ ion.

Identification of dPD and PD were confirmed by LC/ESI-MS, comparing it with predominantly [M+Na]^+^ ion in the positive mode and [M–H]^−^ ion in the negative mode. In order to increase the signal-to-noise, 5 μM of sodium acetate was added to the mobile phase to enhance the molecular ion intensity, and then the significantly increased [M+Na]^+^ ion was presented in the spectrum of ESI-MS. Meanwhile, a high abundance of [M–H]^−^ is observed in the negative mode. In the same manner, peaks at *m/z* 1115.5 and 1247.5 in the positive spectrum and the peaks at *m/z* 1091.6 and 1223.6 in the negative spectrum (Figure 5) were assigned to known platycosides with molecular weight of 1092 and 1224, respectively.

Finally, the structures of dPD and PD were elucidated on the basis of spectroscopic method including UV, IR, and ^13^C-NMR. All the ^13^C-NMR data are in very good accordance to previously published references (data not shown) [27,28].

## 3. Experimental Section

### 3.1. Plant Materials and Chemicals

The roots of *P. grandiflorum* were purchased from Tong Ren Tang drugstore and identified by Professor Yinan Zheng. Its voucher specimen was deposited in College of Chinese Medicinal Material, Jilin Agricultural University. Snailase was purchased from Beijing Biodee Biotechnology Co., Ltd (http://www.biodee.net). β-glucanase, cellulase and amylase were supported by Ningxia Xiasheng Group Co., Ltd. (http://www.sunsonenzymes.com).

HPLC-grade acetonitrile and methanol were purchased from Fisher Chemicals (USA). Other chemicals for extraction and separation were all of analytical grade from Beijing Chemical Factory. Water was purified using a Milli-Q water purification system (Millipore, Bedford, MA, USA).

### 3.2. Sample Preparation

About 5.0 kg of powdered roots of Platycodi Radix was extracted with 70% ethanol by ultrasonic-assisted extraction for 0.5 h with 3 times. The combined extract was evaporated with a rotary evaporator under reduced pressure, suspended in water, and then partitioned with ethyl acetate and *n*-butanol. The *n*-butanol layer (crude platycosides, 85 g) was evaporated, lyophilized, and stored in a desiccator until further use.

### 3.3. Enzymatic Preparation of dPD and PD from Crude Platycosides

Snailase were incubated with crude platycosides in a pH 4.5 sodium acetate buffer with agitation at different temperature (varying reaction temperature from 35 to 45 °C) and different enzyme load (varying from 5 to 20%) for certain time (varying reaction time from 4 to 24 h). The mixtures were subsequently placed in a water bath at 90 °C to terminate the enzymatic reaction. The reaction mixtures were individually evaporated, dissolved in methanol, and filtered through a 0.45 μm nylon filter membrane prior to injection into the HPLC system. The chromatographic peaks of six platycosides were confirmed by comparing their retention time with those of the reference standards. Quantification was carried out by the integration of the peak using external standard method.

### 3.4. HPLC Analysis of Platycosides

**Figure 6:**
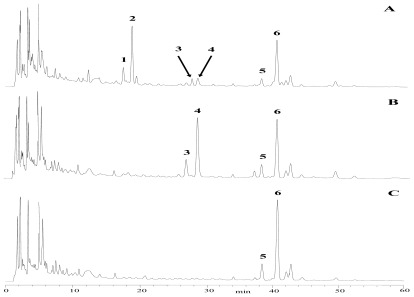
HPLC chromatograms of enzymatic preparation samples for 0 h (**A**), 5 h (**B**), and 24 h (**C**). Column: Hypersil ODS2 (250 mm × 4.6 mm, I.D., 5 μm); mobile phase: acetonitrile-water (Acetonitrile: 0–30 min, 18–22%; 30–60 min, 22–25%); flow rate: 1.0 mL/min; detection wavelength: 210 nm. Key to peak identity: 1, dPE; 2, PE; 3, dPD3; 4, PD3; 5, dPD; 6, PD.

The HPLC analysis was performed with a HPLC instrument (Agilent 1100, USA) equipped with a quaternary solvent delivery system, a column oven and UV detector. A HPLC method was developed using a reversed-phase C18 column (Hypersil ODS2, 250 mm × 4.6 mm I.D., 5 μm). The column temperature was set at 30 °C and detection wavelength was set at 210 nm. The mobile phase was consisted of water (A) and acetonitrile (B) with flow rate of 1.0 mL/min. The gradient elution was programmed as follows: 0–30 min, 18–22% B; 30–60 min, 22–25% B. The sample was dissolved in the methanol with 200 mg/mL of concentration. Then the 20 μL of sample solution was directly injected into the chromatographic column manually. Three typical chromatograms of samples, before and after snailase preparation, are shown in Figure 6.

### 3.5. LC/ESI-MS Analysis

The platycosides were analyzed by a LC/ESI-MS instrument from Shimadzu consisting of a Surveyor MS pump and UV detector. The mass spectrometer was equipped with an electrospray ion (ESI) source operated in positive and negative mode. All of trap analyzer parameters were optimized and set as follow: capillary voltage of 4.5 kV, cone voltage of 45 V, source temperature of 100 °C, desolvation temperature of 250 °C, and N_2_ sheath gas of 90 L/h. The inject volume of sample was 5.0 μL.

### 3.6. Experimental Design

In the present investigation, we employed the software Design Expert (Trial Version 7.1.6, Stat-Ease Inc., Minneapolis, MN) for experimental design, data analysis and model building. A Box-Behnken design (BBD) with three variables was used to determine the response pattern and then to establish a model.

Experimental data were fitted to a quadratic polynomial model and regression coefficient obtained. The non-linear computer-generated quadratic model used in the response surface was as follow:

$$Y=\beta_{0}+\sum_{j=1}^{k}\beta_{j}X_{j}+\sum_{j=1}^{k}\beta_{jj}X_{j}^{2}+\sum \sum_{i<j}\beta_{ij}X_{i}Y_{j}$$

where *Y* is the estimated response, *β*_0_, *β_j_*, *β_jj_* and *β_ij_* are the regression coefficients for intercept, linearity, square and interaction, respectively, while *X_i_*, *X_j_* are the independent coded variables.

### 3.7. Data Analysis

Data were expressed as standard errors of the means (SEM) of three replicated determinations. The response obtained from each set of experimental design (Table 1) was subjected to multiple non-linear regressions using the Design Expert software. The quality of the fit of the polynomial model equation was expressed by the coefficient were checked by *F*-test and *p*-value.

## 4. Conclusions

In this paper, the conditions for enzymatic preparation of deapio-platycodin D and platycodin D from Radix Platycodi were optimized by using RSM and BBD. The results demonstrated that the change of reaction temperature, enzyme load and reaction time could significantly affect the yields of dPD and PD. The estimated models were able to indicate preparation conditions, allowing superior conversion yield. The highest yields predicted for dPD and PD could be attained at optimal conditions including 43 °C of reaction temperature, 15% of enzyme load and 22 h of reaction time. Thus, this methodology could provide an example for a model to examine the non-linear nature between independent variables and responses in a short-term experiment.

## Figures and Tables

**Table 1:** Box-Behnken experimental design with the independent variables.

	Coded variables levels			*Y* dPD and PD (mg/L)	
	
Run	*X*_1_, Reaction temperature (°C)	*X*_2_, Enzyme load (%)	*X*_3_, Reaction time (h)	Actual	Predicted
1	−1 (35)	−1 (5.0)	0 (14)	7.28	7.45
2	1 (45)	−1 (5.0)	0 (14)	12.00	11.46
3	−1 (35)	1 (20)	0 (14)	8.90	9.44
4	1 (45)	1 (20)	0 (14)	13.22	13.05
5	−1 (35)	0 (12.5)	−1 (4)	8.63	8.18
6	1 (45)	0 (12.5)	−1 (4)	11.73	11.98
7	−1 (35)	0 (12.5)	1 (24)	10.92	10.67
8	1 (45)	0 (12.5)	1 (24)	14.02	14.48
9	0 (40)	−1 (5.0)	−1 (4)	9.03	9.32
10	0 (40)	1 (20)	−1 (4)	10.25	10.16
11	0 (40)	−1 (5.0)	1 (24)	10.79	10.87
12	0 (40)	1 (20)	1 (24)	13.89	13.60
13	0 (40)	0 (12.5)	0 (14)	13.75	13.67
14	0 (40)	0 (12.5)	0 (14)	13.08	13.67
15	0 (40)	0 (12.5)	0 (14)	13.89	13.67
16	0 (40)	0 (12.5)	0 (14)	13.89	13.67
17	0 (40)	0 (12.5)	0 (14)	13.75	13.67

**Table 2:** Analysis of variance for the fitted quadratic polynomial model of enzymatic preparation of dPD and PD.

Source	SS	DF	MS	*F*-value	Prob > *F*	
Model	78.13	9	8.68	33.43	<0.0001	significant
Residual	1.82	7	0.26			
Lack of fit	1.36	3	0.45	3.96	0.1086	insignificant
Pure error	0.46	4	0.11			

SS, sum of squares; DF, degree of freedom; MS, mean square.

**Table 3:** Estimated regression model of relationship between response variables (yield of dPD and PD) and independent variables (*X*_1_, *X*_2_, *X*_3_).

Variables	DF	SS	MS	*F*-values	*p*-value
*X*_1_	1	29.02	29.02	111.78	<0.0001
*X*_2_	1	6.39	6.39	24.59	0.0016
*X*_3_	1	12.45	12.45	47.94	0.0002
*X*_1_*X*_2_	1	0.041	0.041	0.16	0.7032
*X*_1_*X*_3_	1	0.000	0.000	0.000	1.0000
*X*_2_*X*_3_	1	0.89	0.89	3.43	0.1064
*X*_1_^2^	1	9.39	9.39	36.17	0.0005
*X*_2_^2^	1	14.11	14.11	54.34	0.0002
*X*_3_^2^	1	3.06	3.06	11.80	0.0109

**Table 4:** Optimum conditions and the predicted and experimental value of response at the optimum conditions.

	Reaction temperature (°C)	Enzyme load (%)	Reaction time (h)	Yield of dPD and PD (mg/mL)
Optimum conditions	43.13	15.00	22.23	14.93 (predicted)
Modified conditions	43	15	22	14.81 (actual)
